# Short- and long-term risks of photoselective laser vaporization of the prostate: a population-based comparison with transurethral resection of the prostate

**DOI:** 10.1080/07853890.2023.2192046

**Published:** 2023-03-28

**Authors:** Alisa Salmivalli, Otto Ettala, Pertti Nurminen, Pekka Kinnala, Peter J. Boström, Ville Kytö

**Affiliations:** a Department of Clinical Research, University of Turku, Turku, Finland; b Department of Urology, Satasairaala Central Hospital, Wellbeing Services County of Satakunta, Pori, Finland; c Department of Urology, Turku University, Turku University Hospital, Turku, Finland; d Heart Center, Turku University Hospital, University of Turku, Turku, Finland; e Turku Clinical Research Center, Turku University Hospital, Turku, Finland; f Research Services, Wellbeing Services County of Southwest Finland, Turku, Finland

**Keywords:** Long-term risks, Oral anticoagulation, Photoselective vaporization of the prostate, PVP, Reoperation, Transurethral resection of the prostate, TURP

## Abstract

**Background:**

Transurethral resection of the prostate (TURP) is the standard surgical treatment for benign prostate enlargement (BPE). Photoselective vaporization of the prostate (PVP) is an alternative, but there is limited real-life evidence of PVP risks.

**Objective:**

To compare short- and long-term risks of PVP to those of TURP in the treatment of BPE.

**Materials and methods:**

Consecutive patients who underwent elective PVP or TURP between 2006 and 2018 in 20 hospitals in Finland were retrospectively studied using a combination of national registries (*n* = 27,408; mean age 71 years). Short-term risks were postoperative mortality, major adverse cardiovascular events (MACE), and reoperations for bleeding. Long-term risks were reoperations for BPE or any urethral operations within 12 years. Differences between treatment groups were balanced by inverse probability of treatment weighting. Risks were analyzed using the Kaplan–Meier method and Cox regression.

**Results:**

There were no differences in postoperative mortality or MACE between the study groups. Reoperations for bleeding were less frequent after PVP (0.9%, HR: 0.72, *p* = 0.042). Bleeding was more likely in patients with atrial fibrillation (number needed to treat [NNT] for PVP vs TURP: 61). Cumulative incidence for reoperation was higher after PVP (23.5%) than after TURP in long-term follow-up (17.8%; HR: 1.20, *p* < 0.0001, NNT: −31.7).

**Conclusions:**

PVP is associated with lower postoperative bleeding risk but higher long-term reoperation risk than TURP. Patients with high bleeding risk and a low likelihood of needing reoperation appear most suitable for laser vaporization.

**KEY MESSAGE**
PVP is associated with lower postoperative bleeding risk but higher long-term reoperation risk than TURP. PVP appears an attractive treatment option, especially for patients with high bleeding risk and a low likelihood of needing a reoperation.

## Introduction

Transurethral resection of the prostate (TURP) is considered the standard operation and a reference technique for the surgical management of lower urinary tract symptoms (LUTS) [1]. However, in recent years, there have been efforts to find safe and effective alternatives to TURP [2]. Despite the growing popularity of laser enucleation techniques, photoselective vaporization of the prostate (PVP) remains a widely used laser technique, but the evidence for the safety and long-term efficacy of PVP, compared to TURP, is limited, especially in patients at high risk due to oral anticoagulation treatment.

Long-term follow-up data for PVP is mainly available 3 or 5 years after the primary operation, and the estimates of reoperation rates vary widely [3–7], although the rates after PVP (11–33%) have been found to be higher than after TURP (1.8–3.0%) [7,8]. According to current guidelines, short-term results of the 80-W laser and mid-term results of the 120-W laser are comparable to TURP, but there remains a lack of long-term randomized controlled trials (RCT) [1].

Along with other laser modalities, PVP is a tempting alternative to TURP, with better intraoperative bleeding control and a supposedly lower risk of postoperative bleeding complications, which are major concerns when operating on at-risk patients [1]. However, PVP’s apparently superior safety is based mostly on small patient-series studies with limited evidence, in contrast to studies of TURP for high-risk patients [1,9]. The purpose of this study was to compare the short- and long-term risks of PVP and TURP on a national level.

## Material and methods

### Design

**Figure 1.**
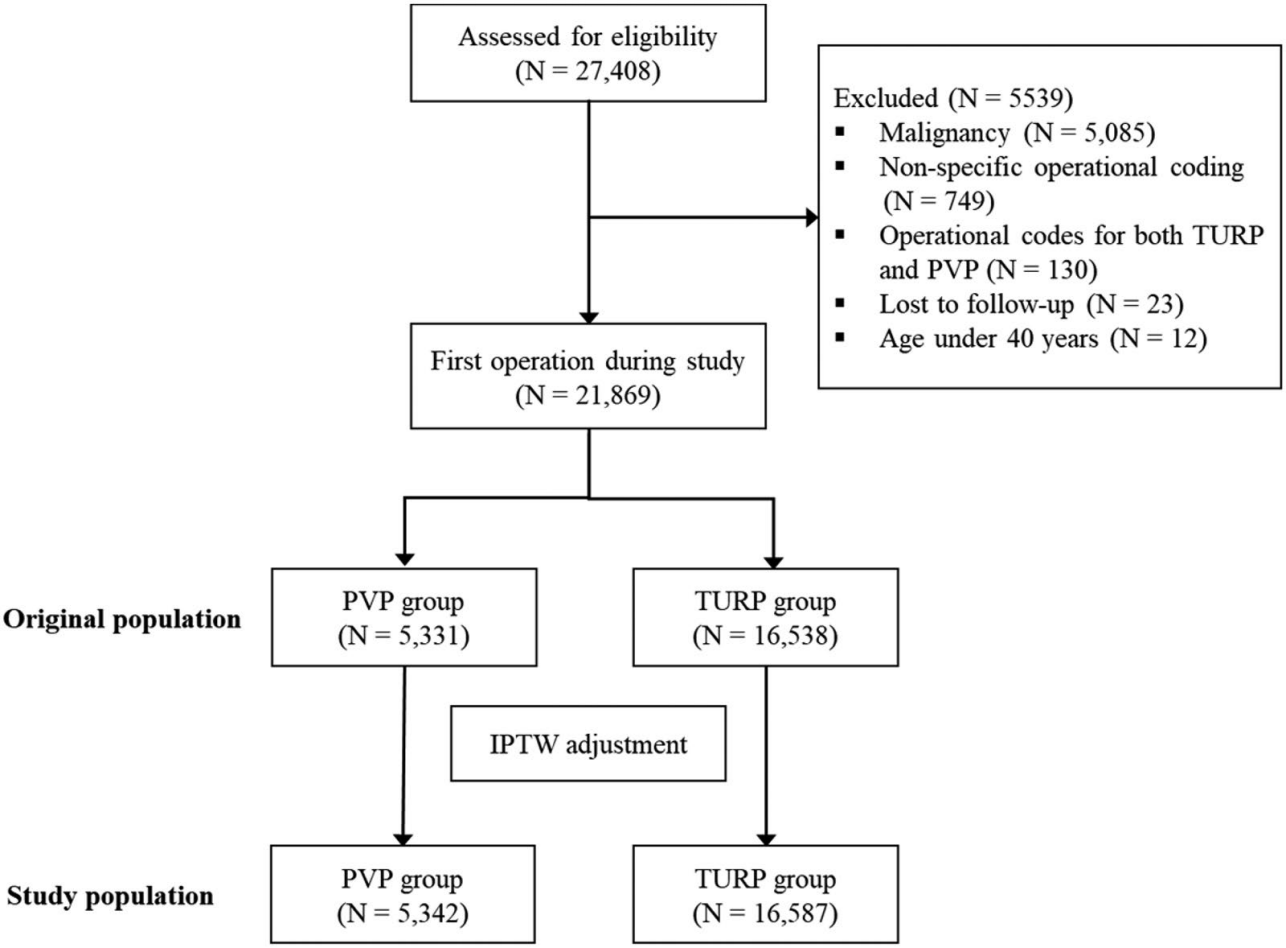
Study flowchart.

The data for all consecutive BPE patients undergoing elective PVP or TURP procedures during 1 January 2006 and 30 September 2018 in all Finnish institutions that performed both procedures were retrospectively collected from the Care Register for Healthcare in Finland (CRHF). All three generations of PVP, and both bipolar and monopolar TURP were represented in the data. Only the first operation for each patient during the study period was included, and there was a two-year ‘washout’ period prior to the study (i.e. those who underwent prostate operations in 2004–2005 were excluded). Patients with urological or genital malignancy, with a non-specific tumor of the urinary tract, with operational codes for both PVP and TURP or otherwise non-specific operational coding at the time of the index procedure, under 40 years of age, or lost to follow-up were excluded (Figure 1).

Short-term risks were postoperative mortality, major adverse cardiovascular events (MACE; defined as myocardial infarction, stroke, or cardiovascular death), and reoperation for bleeding within 90 days of the index operation. Long-term risks were reoperations from BPE or any urethral operations within 12 years. Risks were determined using The Nordic Medico-Statistical Committee Classification of Surgical Procedures (NCPS) coding used nationally in Finland and ICD-10 coding in the CRHF. Comorbidities were determined using ICD-10 coding, as previously described [10]. Risks are defined in Supplementary Table 1. Follow-up ended on 31 December 2018.

**Table 1.** Baseline features of benign prostate hyperplasia patients treated with laser or TURP. Features of all patients and inverse probability weight-balanced cohort. SMD = standardized mean difference.

Variable	Original study population				Weighted study population			
	PVP	TURP			PVP	TURP		
	*n* = 5331	*n* = 16,538	*P*-value	|SMD|	*n* = 5,342	*n* = 16,587	*P*-value	|SMD|
Age, years (SD)	70.3 (8.6)	70.6 (8.5)	0.008	0.042	70.5 (8.5)	70.5 (8.7)	0.790	0.004
AIDS/HIV	0.1%	0.1%	0.106	0.023	0.1%	0.1%	0.973	0.001
Alcohol abuse	2.0%	2.6%	0.020	0.038	2.5%	2.5%	0.906	0.002
Anemia	1.3%	1.5%	0.356	0.015	1.4%	1.4%	0.856	0.003
Atrial fibrillation	17.7%	12.1%	<0.0001	0.159	13.5%	13.5%	0.995	0.0001
Cerebrovascular disease	11.7%	9.6%	<0.0001	0.070	10.0%	10.1%	0.770	0.005
Chronic pulmonary disease	8.4%	8.8%	0.354	0.015	8.4%	8.7%	0.557	0.009
Coagulopathy	0.6%	0.4%	0.085	0.026	0.4%	0.4%	0.919	0.002
Dementia	3.1%	3.0%	0.817	0.004	3.1%	3.0%	0.810	0.004
Diabetes	12.2%	11.6%	0.213	0.020	11.6%	11.7%	0.802	0.004
Heart failure	8.1%	6.4%	< 0.0001	0.064	6.8%	6.8%	0.837	0.003
Heart valve disease	5.8%	4.1%	< 0.0001	0.078	4.6%	4.6%	0.972	0.001
Hypertension	27.9%	25.6%	0.001	0.052	25.8%	26.1%	0.670	0.007
Liver disease	1.0%	0.9%	0.607	0.008	0.9%	0.9%	0.984	0.0003
Neurological disease*	4.6%	4.8%	0.170	0.022	4.7%	4.6%	0.779	0.004
Cancer**	8.3%	8.2%	0.840	0.003	8.1%	8.2%	0.684	0.006
Metastatic	0.2%	0.3%	0.906	0.002	0.2%	0.3%	0.934	0.001
Paralysis	0.3%	0.6%	0.021	0.039	0.5%	0.5%	0.555	0.009
Peripheral vascular disease	5.5%	4.7%	0.011	0.039	4.8%	4.9%	0.745	0.005
Prior myocardial infarction	7.0%	5.9%	0.004	0.044	6.3%	6.2%	0.719	0.005
Psychotic disorder	0.8%	1.3%	0.001	0.054	1.2%	1.2%	0.990	0.0002
Rheumatic disease	2.5%	2.9%	0.146	0.023	2.8%	2.8%	0.925	0.001
Renal failure	2.7%	3.0%	0.276	0.017	2.9%	2.9%	0.940	0.001
University hospital***	51.3%	43.5%	< 0.0001	0.157	44.5%	44.5%	0.293	0.017
Year of operation			< 0.0001	0.246			0.954	0.009

* Other than cerebrovascular disease

** Patients with urogenital cancer were excluded

*** As the surgical center.

The inverse probability of treatment weight (IPTW) was used to balance baseline differences between the treatment groups [11]. Propensity scores based on age, AIDS/HIV, alcohol abuse, anemia, atrial fibrillation, cerebrovascular disease, chronic pulmonary disease, coagulopathy, dementia, diabetes, heart failure, heart valve disease, hypertension, liver disease, non-prostate cancer, metastatic cancer, paralysis, peripheral vascular disease, prostate cancer, prior myocardial infarction, psychotic disorder, rheumatic disease, renal failure, place of procedure (high volume vs. intermediate or low volume center), and calendar year of operation (Table 1) were created using logistic regression and were used for the IPTW calculations. To improve balancing, the IPTW was stabilized, resulting in treatment weights ranging from 0.47 to 2.96 [11], and adjustment with the IPTW resulted in balanced study groups (Table 1). Subgroup analyses, with separate propensity scoring and IPTW adjustments, were performed for patients aged below 70 years and those aged 70 years or above and for patients with and without atrial fibrillation (AF). Baseline variables were balanced between the treatment groups in all subgroups (*p* > 0.327 and standardized mean difference [SMD] < 0.028 for all).

### Data sources

CRHF registry data, including data for all hospital admissions and major surgical procedures and cancer data from the Finnish Cancer Registry, were obtained from the National Institute for Health and Welfare of Finland (permission no: THL/2245/5.05.00/2019). Mortality data were obtained from Statistics Finland (TK-53-484-20). These registries are mandated by law and cover the entire Finnish population.

### Ethical considerations

This study was approved by the National Institute for Health and Welfare of Finland (permission no: THL/2245/5.05.00/2019) and Statistics Finland (TK-53-484-20). The legal basis for processing personal data was public interest and scientific research (EU General Data Protection Regulation 2016/679, Article 6(1)(e) and Article 9(2)(j); Data Protection Act, Sections 4 and 6). Due to the retrospective study design, informed consent was not required, and the participants were not contacted.

### Statistical analysis

Differences between the groups were analyzed with chi-squared, Jonckheere–Terpstra, and t-tests. The effect sizes of the baseline characteristics for the groups were evaluated by SMD, and risks were examined using the Kaplan–Meier method and Cox regression. Proportional hazard assumptions were confirmed with Schoenfeld residuals. Patients were censored at the time of death when studying other risks. Regression models were weighted with stabilized IPTW, and the number needed to treat (NNT) was calculated as previously described [12]. The extent of unmeasured confounds was estimated using the E-value [13]. Results are presented as means, medians, percentages, SMDs, or hazard ratios (HR) with 95% confidence intervals (CI), interquartile ranges (IQR), or standard deviations (SD). Statistical significance was defined as a p-value below 0.05. Analyses were performed with SAS version 9.4 (SAS Institute, Inc., Cary, NC, USA).

## Results

A total of 27,408 procedures were performed in 20 operating centers between 2006 and 2018. After exclusions based on the criteria described above, 5331 patients treated with PVP and 16,538 patients treated with TURP were included (Figure 1). The conversion rate from PVP to TURP was 2.2%.

The baseline features of the original population and the weighted study population are presented in Table 1. In the original population, patients treated with PVP had a higher frequency of AF, vascular disease, and heart failure than those treated with TURP in the study population; PVP-treated patients were also slightly younger. The baseline differences between the PVP and TURP groups were balanced with IPTW adjustment, resulting in a study population of 5,342 PVP and 16,587 TURP patients.

The short-term risks are presented in Table 2; 90-day postoperative mortality for all operated patients was 0.6% (125 patients), resulting from 0.4% mortality for PVP and 0.6% for TURP. A total of 427 patients had MACE, and 256 were operated upon due to bleeding within 90 days from the index procedure. There were no statistically significant differences in the occurrence of 90-day postoperative mortality or MACE between the study groups, but reoperation for bleeding was less frequent after PVP than after TURP (HR: 0.72; CI: 0.53–0.99; *p* = 0.042). In subgroup analyses, older (≥ 70 years) patients and those with AF presented with higher rates of reoperation because of bleeding than did younger men and those without AF.

**Table 2.** Short-term postoperative mortality, MACE, and major bleeding rates for benign prostate hyperplasia patients treated with PVP or TURP (reference group).

Short-term risks	Adjusted %		Adjusted HR		
	PVP	TURP	HR (95%CI)	*P*-value	NNT
Postoperative mortality					
All patients	0.4%	0.6%	0.72 (0.46–1.12)	0.144	583.0
<70 years	0.1%	0.2%	0.48 (0.14–1.61)	0.235	782.2
≥70 years	0.7%	0.9%	0.75 (0.46–1.24)	0.263	434.9
Atrial fibrillation	0.2%	0.1%	1.15 (0.61–2.18)	0.660	−497.7
No atrial fibrillation	0.3%	0.5%	0.54 (0.28–1.01)	0.051	431.1
MACE					
All patients	1.8%	2.0%	0.87 (0.69–1.10)	0.253	386.1
<70 years	0.9%	1.1%	0.86 (0.54–1.35)	0.503	664.5
≥70 years	2.5%	2.8%	0.88 (0.67–1.15)	0.337	301.4
Atrial fibrillation	3.5%	4.3%	0.83 (0.55–1.23)	0.351	140.6
No atrial fibrillation	1.5%	1.7%	0.90 (0.69–1.18)	0.440	599.8
Reoperation due to bleeding					
All patients	0.9%	1.3%	0.72 (0.53–0.99)	0.042	284.8
<70 years	1.1%	1.3%	0.83 (0.55–1.26)	0.379	441.4
≥70 years	0.7%	1.2%	0.58 (0.36–0.94)	0.028	199.1
Atrial fibrillation	1.3%	2.9%	0.44 (0.24–0.81)	0.009	61.1
No atrial fibrillation	0.9%	1.0%	0.86 (0.60–1.23)	0.411	721.0

HR: Hazard ratio; NNT: number needed to treat.

**Figure 2.**
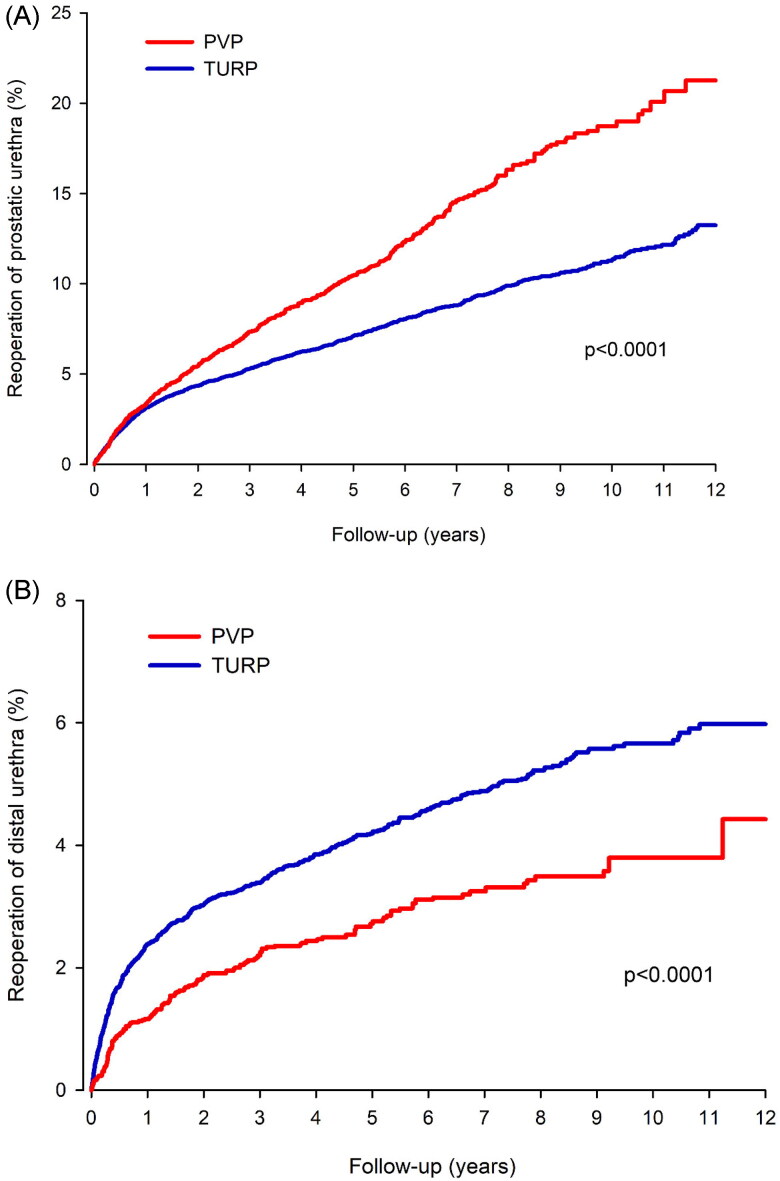
(A) Cumulative incidence of prostatic urethra reoperation after photoselective vaporization of the prostate (PVP) and transurethral resection of the prostate (TURP). (B) Cumulative incidence of distal urethra reoperation after photoselective vaporization of the prostate (PVP) and transurethral resection of the prostate (TURP).

The long-term risks are presented in Table 3. The median follow-up during the 12-year cumulative period was 4.3 years; overall, total reoperation rates were higher after PVP than after TURP, irrespective of age or the presence of AF. The higher reoperation rate after PVP is attributed mostly to a higher rate of prostatic urethra reoperations, such as resection or vaporization of residual adenoma or discission of the bladder neck stricture (Figure 2(A)). In contrast, there were fewer reoperations of the distal urethra after PVP than after TURP (Figure 2(B)). The E-value for any reoperation in the overall cohort was 1.69 (1.40–1.95).

**Table 3.** Long-term (12-year) reoperations for benign prostate hyperplasia patients treated with PVP or TURP (reference group).

Long-term risks	Adjusted %		Adjusted HR		
	PVP	TURP	HR (95% CI)	*P*-value	NNT
Reoperation, overall					
All patients	23.5%	17.8%	1.20 (1.09–1.31)	<0.0001	−−31.7
<70 years	25.3%	17.3%	1.29 (1.14–1.47)	<0.0001	−22.5
≥70 years	19.4%	17.8%	1.31 (1.00–1.39)	0.058	−20.6
Atrial fibrillation	31.0%	16.3%	1.69 (1.34–2.14)	<0.0001	−10.3
No atrial fibrillation	22.8%	17.9%	1.15 (1.04–1.27)	0.006	−41.8
Reoperation of prostatic urethra					
All patients	21.3%	13.2%	1.66 (1.41–1.72)	<0.0001	−12.9
<70 years	23.2%	13.4%	1.67 (1.45–1.92)	<0.0001	−12.6
≥70 years	17.2%	12.6%	1.48 (1.28–1.71)	<0.0001	−18.3
Atrial fibrillation	28.5%	11.2%	2.24 (1.72–2.93)	<0.0001	−8.2
No atrial fibrillation	20.7%	13.4%	1.49 (1.34–1.66)	<0.0001	−17.0
Reoperation of distal urethra					
All patients	4.4%	6.0%	0.64 (0.53–0.77)	<0.0001	47.4
<70 years	4.5%	5.2%	0.66 (0.51–0.86)	0.002	57.6
≥70 years	4.2%	6.9%	0.63 (0.49–0.81)	0.0003	40.0
Atrial fibrillation	6.3%	6.9%	1.07 (0.70–1.63)	0.760	−216.4
No atrial fibrillation	4.2%	6.0%	0.60 (0.49–0.73)	<0.0001	42.6

HR: Hazard ratio; NNT: number needed to treat.

## Discussion

In this population-based study of over 20,000 operated patients, we found that short-term bleeding risk is lower and long-term reoperation risk is higher after PVP than after TURP. The current knowledge of the safety of PVP in patients at high risk of bleeding is based mostly on case series, since such patients are often excluded from RCTs [1]. The dilemma is that urologic surgery is generally categorized, in procedural bleeding risk stratification, as high risk [14,15], and therefore the oral anticoagulation treatment that is used mainly to prevent thromboembolism in AF may need to be ceased [16]. Transurethral resection syndrome and irrigation fluid absorption remain also significant risks of aforesaid operations [17]. These adverse events were not considered in the current study, but should be kept in mind, when planning an operation for BPE.

In our large national series of consecutive operated BPE patients, we found no difference in the risk of short-term postoperative mortality or MACE between PVP and TURP. However, PVP was associated with a lower risk of reoperation for bleeding than TURP, with an HR of 0.72 (*p* = 0.042) and an NNT of 285 in the overall study population. The impact of PVP in attenuating bleeding risk was more prominent in patients with AF, with an NNT of 61. These results indicate that PVP is most beneficial for patients at high risk of bleeding, especially those being treated with oral anticoagulants.

In the current study, there was no difference in the risk of 90-day postoperative mortality between PVP and TURP. Gilfrich et al. had similar results in their large study of 95,577 cases: 30-day postoperative mortality was 0.58% after PVP and 0.32% after TURP, and the difference disappeared in multivariable analysis [18]. Estimates of postoperative mortality were also similar in a study by Bhojani et al.: 0.4% after TURP and 0.3% after PVP [19]. They also found no significant difference in overall complications or perioperative mortality between TURP and PVP [19]. Interestingly, the incidence of MACE was surprisingly low in their study: 0.3% after TURP and 0.2% after PVP [19], compared to 2.0% after TURP and 1.8% after PVP in the current study. This might be explained by differences in the design: Bhojani et al. analyzed complications occurring 30 days postoperatively, while in the current study, the time frame for short-term complications was 90 days; the current study also included cardiovascular deaths among the risks, and there might have been differences in the patient exclusion/inclusion criteria that affected the results.

The adjusted rate of reoperations for bleeding was 0.9% after PVP vs. 1.3% after TURP in our overall cohort, and 1.3% after PVP vs. 2.9% after TURP in patients with AF. In the existing literature, postoperative bleeding after TURP is a well-known complication [3], and perioperative oral anticoagulation treatment increases this risk [20], but there is also a risk of postoperative bleeding after PVP [20]. The overall rate of reoperations for bleeding in our study is somewhat higher than those reported previously, reflecting the real-life, all-comer design. In contrast, Reich et al. observed no postoperative bleeding after PVP in their study of 66 high-risk patients (26 taking oral anticoagulants) [21], and Ruszat et al. and Sandhu et al. similarly observed no bleeding complications that necessitated blood transfusions or operations after PVP in their studies of 116 and 24 high-risk patients, respectively [22,23]. In the existing literature, bleeding complications are more common after TURP than after PVP [7,20,24], but major bleeding during PVP may lead to impaired visibility and thus conversion to TURP. Other reasons for conversion are early fibre defects and, with large prostates, insufficient de-obstruction with primary fibre [6]. The conversion rate in the current study was 2.2%, which is lower than the range of the existing data for conversions: 3.5%–5.2% [6,24].

Although PVP seems to be superior to TURP in terms of postoperative bleeding, the downside is the higher proportion of patients needing a later reoperation. The general perception is that prostatic reoperations are necessary more frequently after PVP, but long-term follow-up data are lacking [1]. In a recent study by Elshal et al. the reoperation rate of the prostatic urethra was slightly in favour of PVP at 3 years of follow-up—6.7% after PVP and 9.7% after TURP [5]—whereas in a study by Mordasini et al. the reoperation rates after 5-year follow-up were 14.3% after PVP and 11.9% after TURP [4]. Although no previous comparative data exist beyond 5 years, these results are in line with the current study. It seems that, in the short term, the efficacy of PVP and TURP are similar, but at 5 years, those who initially underwent PVP are reoperated more frequently. More importantly, our data clearly show that the difference becomes more pronounced during longer follow-up. At 12 years, 24% of patients who underwent PVP were reoperated upon compared to 18% of those who underwent TURP.

It is reasonable to argue that the increased rate of reoperations of the prostatic urethra after PVP might be due to the majority of patients in all the studies—including ours—being operated upon using earlier generations of PVP equipment, resulting in poorer reductions in the volume of the prostate [25,26]. In fact, it has been shown that the volume reduction of the new generation 180-W laser system is not inferior to TURP and results in comparable functional results [27,28]. When it comes to the occurrence of clinically significant distal urethral strictures requiring an operative treatment, our data suggest that PVP is superior to TURP, which might be explained by PVP instruments having a smaller diameter than those used in TURP. Further studies on the subject are nevertheless needed. Interestingly, in our study, the advantage of PVP for distal urethral strictures was not evident in patients with AF. The reason for this remains unclear, since patients with OA seem to have similar operation times to control groups in the existing literature [20,22], and one therefore cannot argue that longer operative times or greater bleeding during the operation in patients with AF could be associated with the risk of developing strictures. New endoscopic enucleation techniques have come to replace PVP and TURP to a large extent, as an effort to resolve the relatively high demand for reoperation after aforementioned operations. Enucleation techniques seem safe, but there is still limited evidence of their safety with anticoagulated patients [1]. Further studies should aim to prove the safety and efficiency of endoscopic enucleation techniques with anticoagulated patients, in comparison to previous standard methods like PVP and TURP.

There are several strengths and limitations to our study. The major limitation is the retrospective nature, which leads to several shortcomings. First, due to the nature of NCPS coding, we were unable to differentiate the three generations of PVP into subgroups. Second, we were unable to differentiate bipolar and monopolar TURP. Third, due to a lack of more detailed patient data, we were unable to identify the pretreatment size of the prostate, the surgeon´s level of experience, and varying PVP and TURP techniques, which may have influenced the results. Coding errors could also have occurred and underreporting of medical history data is possible. However, it should be noted that we used a combination of nationwide, previously validated, mandatory-by-law registries [29] and conducted follow-ups for up to 12 years. The analyses were also adjusted for a broad range of potential confounds. Propensity scoring and IPTW, which we used, are among the strongest methods for controlling confounding factors in comparative registry studies. This methodology allows the straightforward presentation of results, similar to randomized trials, while providing superior—or at least non-inferior—bias reduction compared to multivariable regression [30]. The advantage of IPTW compared to matching is the usage of all available data for analysis, although it may be more sensitive to misspecification of the propensity model and extreme propensity-score values [11,30]. In our analyses, we used stabilized IPTW-values to limit the influence of extreme propensity scores. Non-recognized residual confounding is nevertheless possible. Based on the E-value, the observed higher reoperation rate after PVP could be explained by unmeasured confounds associated with both treatment modality and the need for reoperation with risk ratios of 1.7 or more each, above and beyond the measured confounds, but not by weaker confounds [13].

## Supplementary Material

Supplemental Material

## Data Availability

The data that support the findings of this study were obtained from the National Institute for Health and Welfare of Finland (permission no: THL/2245/5.05.00/2019). Mortality data were obtained from Statistics Finland (TK-53-484-20). Restrictions apply to the availability of these data, which were used under license for this study. Data are available from the authors with the permission of National Institute for Health and Welfare and Statistics Finland.
