# Supplementary material for: Short- and long-term risks of photoselective laser vaporization of the prostate: a population-based comparison with transurethral resection of the prostate
Source: Ann Med. 2023 Mar 28;55(1):1287–94. doi: 10.1080/07853890.2023.2192046 (PMC10054157; doi:10.1080/07853890.2023.2192046)
Supplement: Supplemental Material [file IANN_A_2192046_SM8196.docx]

**Supplementary Table 1: Outcome definitions**

| **Outcome definitions** | | |
| --- | --- | --- |
| **Short-term outcomes** | | **Definition** |
| All-cause mortality | | Death by any cause |
| Major adverse cardiovascular event | | Myocardial infarctation, stroke, or cardiovascular death |
|  | Myocardial infarctation | ICD-10 code I21* or I22* |
|  | Stroke | ICD-10 code I60-I64* |
|  | Cardiovascular death | Death with ICD-10 code I* as the underlying cause of death |
| Reoperation for bleeding | | Operation codes KCV22, KCW02, KE2BT, KEV00, KEV02, KWD00 |
| **Long-term outcomes** | | **Definition** |
| Reoperation | | Operation codes KED*, ICD-10 code N32.0 |
| Reoperation of distal urethra | | Operation codes KDV12, KDV10, ICD-10 codes N35.9, N35.8 |
